# Supplementary material for: Treatment of Erythroid Precursor Cells from β-Thalassemia Patients with Cinchona Alkaloids: Induction of Fetal Hemoglobin Production
Source: Int J Mol Sci. 2021 Dec 14;22(24):13433. doi: 10.3390/ijms222413433 (PMC8706579; doi:10.3390/ijms222413433)
Supplement: Supplementary file 1 [file ijms-22-13433-s001.zip › ijms-1428497-supplementary.pdf]

Treatment of erythroid precursor cells from β-thalassemia patients with cinchona alkaloids: induction of fetal hemoglobin production

Zuccato C.[1], Cosenza L.C.[1], Zurlo M.[1], Lampronti I.[1,2], Borgatti M.[1,2], Chiara Scapoli[3], Gambari R.[1,2,4,*], Finotti A.[1,2,4,*]

[1]Department of Life Sciences and Biotechnology, Section of Biochemistry and Molecular Biology, University of Ferrara, 44121, Ferrara, Italy

[2]Thal-LAB, Research Laboratory "Elio Zago" on the Pharmacologic and Pharmacogenomic Therapy of Thalassemia; University of Ferrara, 44121, Ferrara, Italy

[3]Department of Life Sciences and Biotechnology, Section of Biology and Evolution, University of Ferrara, 44121, Ferrara, Italy

[4]Interuniversity Consortium for Biotechnology (C.I.B.), 34148 Trieste, Italy

*Corresponding authors:

Alessia Finotti, Department of Life Sciences and Biotechnology, Section of Biochemistry and Molecular Biology, Ferrara University, Italy. E-mail: alessia.finotti@unife.it

Roberto Gambari, Department of Life Sciences and Biotechnology, Section of Biochemistry and Molecular Biology, Ferrara University, Italy. E-mail: gam@unife.it

SUPPLEMENTARY MATERIALS

Supplementary Figures

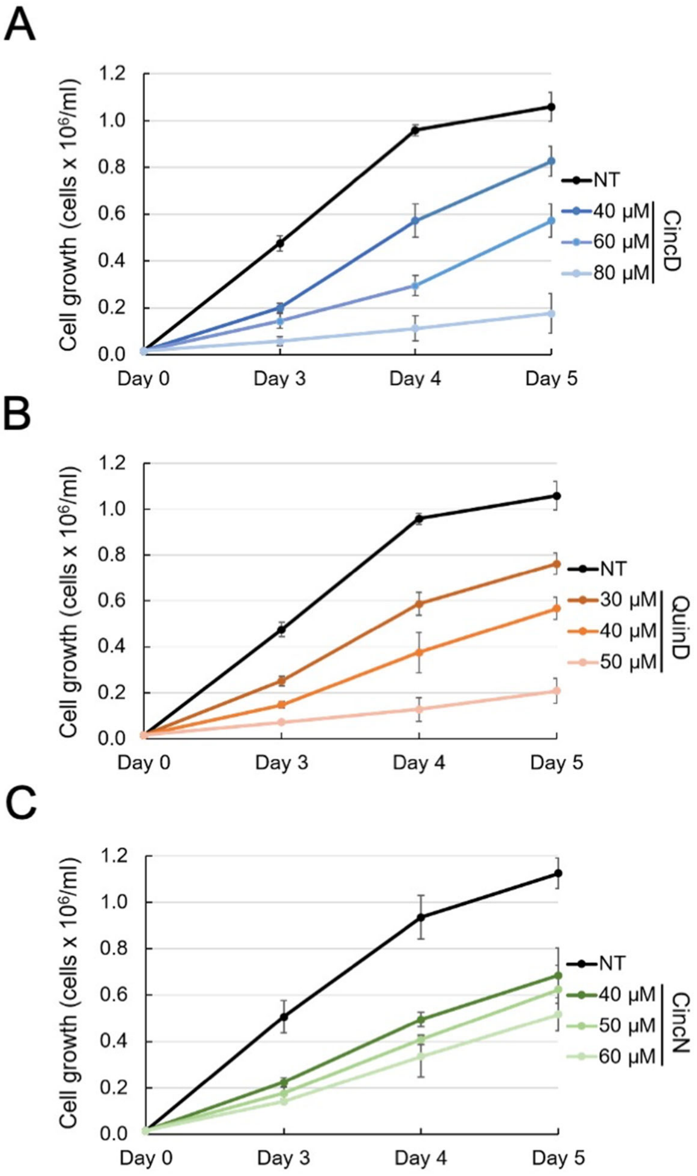

Figure S1. Effects of cinchonidine, quinidine and cinchonine on in vitro proliferation of K562 cells. K562 cells have been cultured in the absence or in the presence of the indicated concentrations of cinchonidine (**A**), quinidine (**B**) and cinchonine (**C**) and the number of cells/ml was determined using a Coulter Counter. NT = untreated cells.

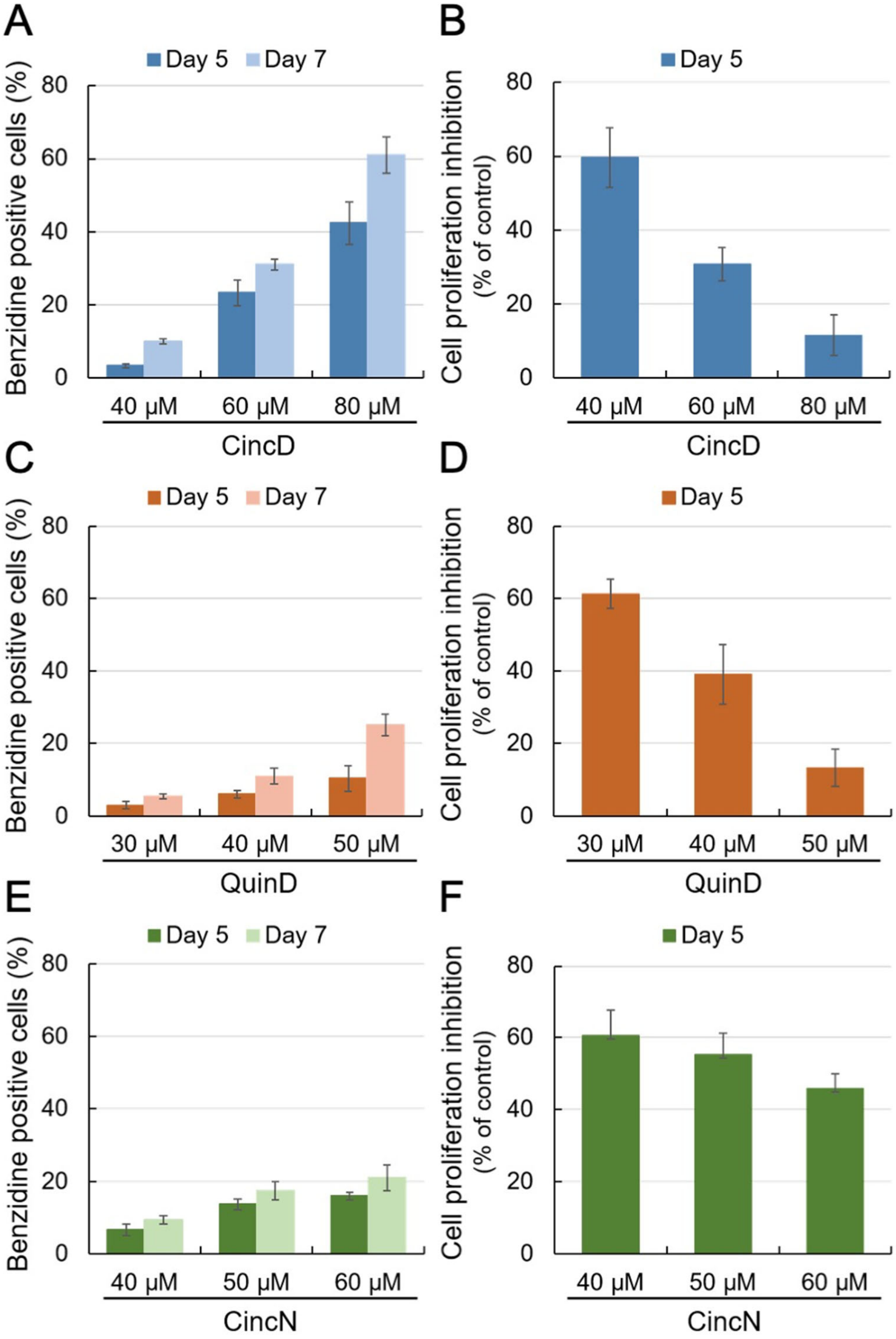

Figure S2. Effects of cinchonidine, quinidine and cinchonine on in vitro differentiation and proliferation of K562 cells. K562 cells have been cultured in the absence or in the presence of the indicated concentrations of of cinchonidine (CincD) (**A,B**), quinidine (QuinD) (**C,D**) and cinchonine (CincN) (**E,F**). After 5 and 7 days the proportion of benzidine positive cells (**A,C,E**) and the number of cells/ml (**B,D,F**) were determined as indicated. The proportion of benzidine-positive cells in control untreated cultures never exceeded 2%.

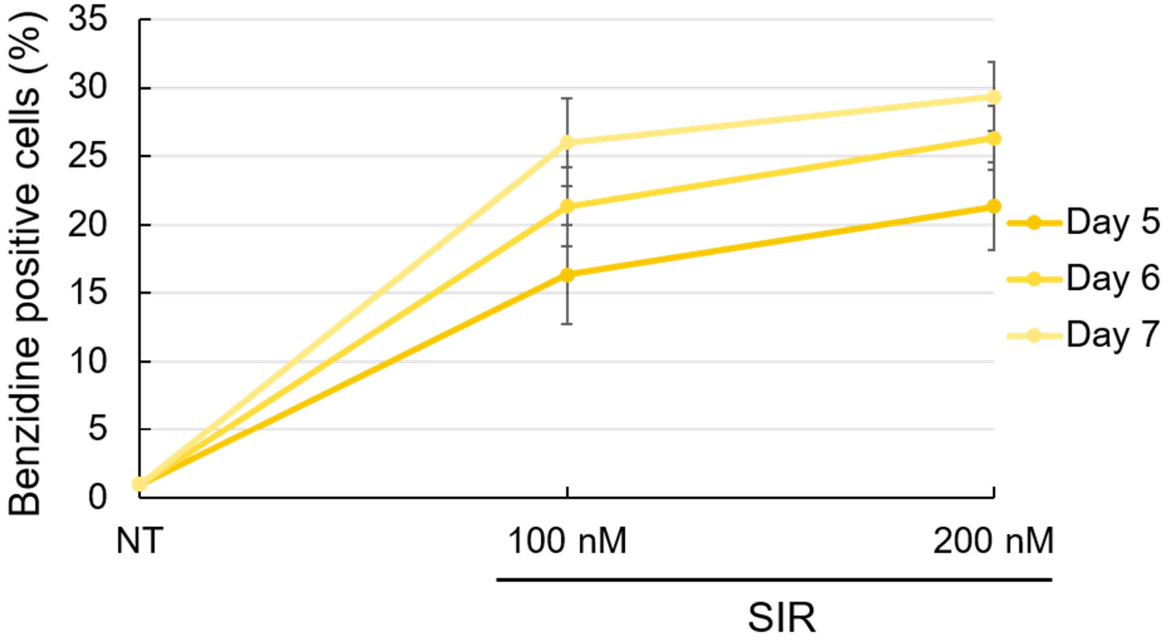

Figure S3. Effects of sirolimus on in vitro differentiation of K562 cells. K562 cells have been cultured in the absence or in the presence of the indicated concentrations of sirolimus for the indicated length of time (days) and the proportion of benzidine-positive cells determined. NT = untreated cells.

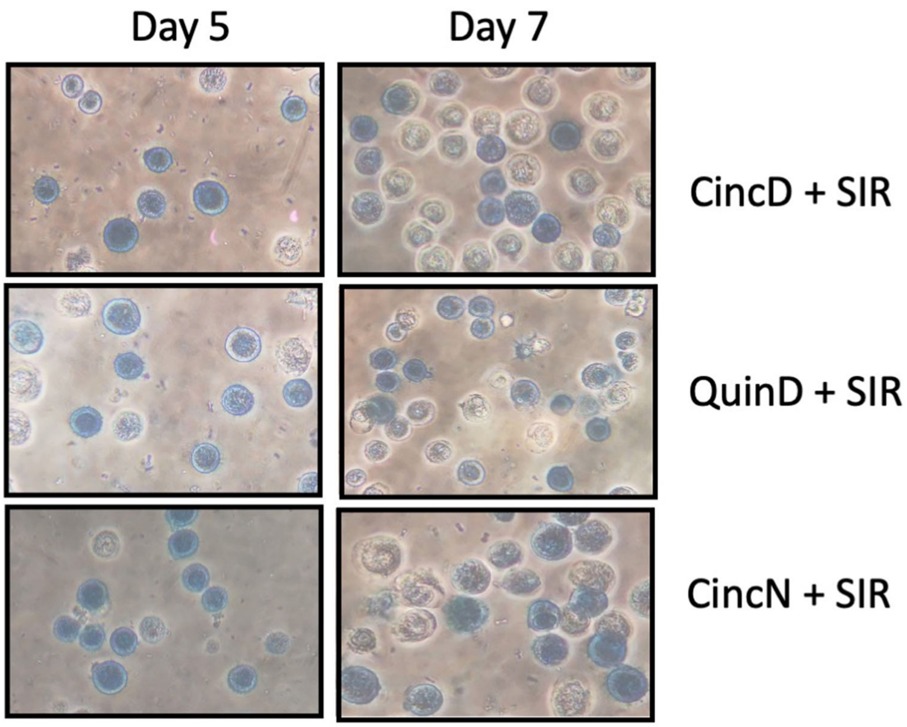

Figure S4. Benzidine positive cells in K562 cell cultures treated for 5 and 7 days with CincD + SIR, QuinD + SIR and CincN + SIR combinations. SIR was used at 200 nM; CincD, QuinD and CincN at 40 μM. Magnitude: 40×.

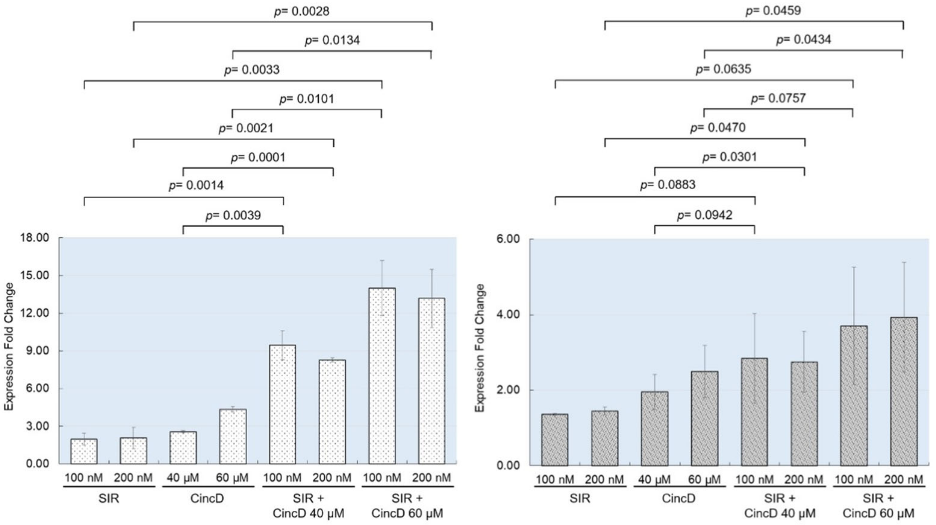

Figure S5. Effects of cinchonidine on *α-globin* (left) and *γ-globin* (right) mRNAs. K562 cells were cultured with the indicated concentrations of SIR, CincD, CincD plus SIR. After 5 days of treatment, RNA was isolated and RT-qPCR performed using primers amplifying *α-globin* (left) and *γ-globin* (right) sequences. The increase of expression of *α-globin* and *γ-globin* mRNAs is presented as fold change in respect to control untreated cells. The data represent the average ± S.E.M. (*n* = 3).

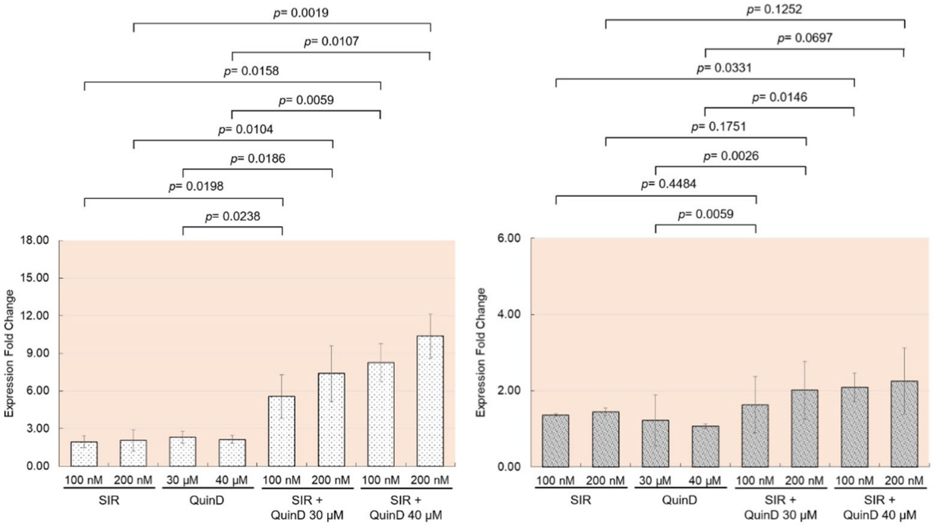

Figure S6. Effects of quinidine on *α-globin* (left) and *γ-globin* (right) mRNAs. K562 cells were cultured with the indicated concentrations of SIR, QuinD, QuinD plus SIR. After 5 days of treatment, RNA was isolated and RT-qPCR performed using primers amplifying *α-globin* (left) and *γ-globin* (right) sequences. The increase of expression of *α-globin* and *γ-globin* mRNAs is presented as fold change in respect to control untreated cells. The data represent the average ± S.E.M.. (*n* = 3).

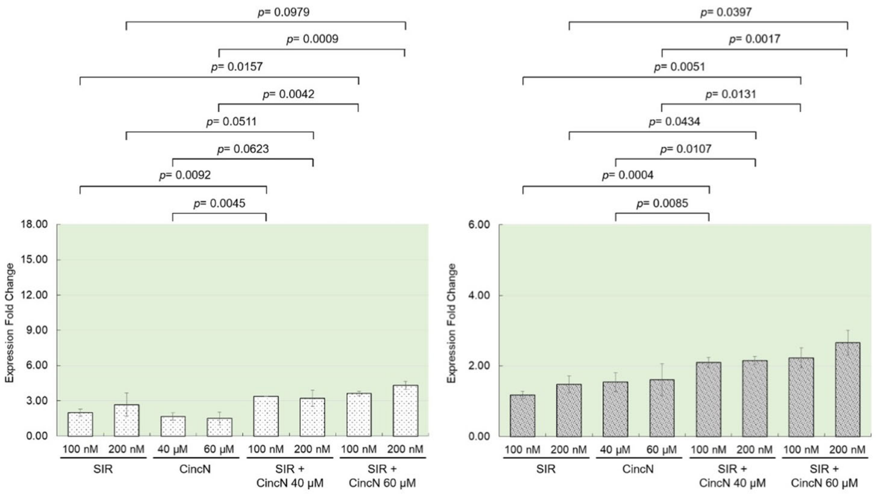

Figure S7. Effects of cinchonine on *α-globin* (left) and *γ-globin* (right) mRNAs. K562 cells were cultured with the indicated concentrations of SIR, CincN, CincN plus SIR. After 5 days of treatment, RNA was isolated and RT-qPCR performed using primers amplifying *α-globin* (left) and *γ-globin* (right) sequences. The increase of expression of *α-globin* and *γ-globin* mRNAs is presented as fold change in respect to control untreated cells. The data represent the average ± S.E.M. (*n* = 3).

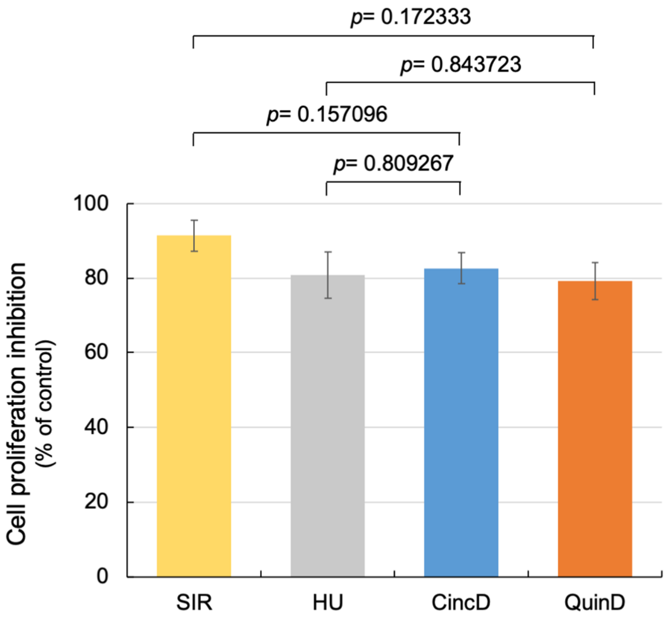

Figure S8. Effects of sirolimus, hydroxyurea, cinchonidine and quinidine on in vitro proliferation of ErPCs from β-thalassemia patients. ErPCs were treated as described in the main text with 100 nM sirolimus (SIR), 100 μM hyrdroxyurea (HU), 60 μM cinchonidine (CincD) and 30 μM quinidine (QuinD). The results are expressed as mean ± S.E.M. of cell number/ml with respect to control untreated cells (= 100) (10 independent experiments performed).

Supplementary Tables

Supplementary Table S1. Effects of cinchonidine (CincD) and quinidine (QuinD) on fetal hemoglobin (HbF) produced by ErPCs from β-thalassemia patients

patients	genotype	HbF (% of total hemoglobins produced)				
		NT	SIR	CincD	QuinD	HU
1	$\beta^0$39/ β^+IVSI-110	34.82	40.27	36.70	38.14	37.94
2	$\beta^0$39/ β^+IVSI-110	26.52	30.93	28.17	30.70	29.97
3	β^+IVSI-110/ β^+IVSI-110	14.29	14.16	13.29	15.17	15.19
4	$\beta^0$39/ β^+IVSI-110	27.47	34.96	32.19	39.49	33.62
5	$\beta^0$39/ $\beta^0$39	30.47	42.18	40.57	45.50	37.22
6	$\beta^0$39/ $\beta^0$39	64.88	72.60	71.26	73.10	67.08
7	$\beta^0$39/ β^+IVSI-110	19.27	26.03	22.32	23.34	25.87
8	β^+IVSI-110/ β^+IVSI-110	8.32	13.40	12.20	15.06	12.61
9	$\beta^0$39/ $\beta^0$39	30.73	34.54	36.81	42.47	31.29
10	$\beta^0$39/ β^+IVSI-110	12.75	9.83	21.62	27.80	11.65

NT = untreated ErPCs; SIR, sirolimus; HU, hydroxyurea. Experimental conditions are reported in Figure 6.

Supplementary Table S2. Effects of combined treatments on fetal hemoglobin (HbF) produced by ErPCs from β-thalassemia patients

patients	genotype	HbF (% of total hemoglobins produced)						
		NT	SIR	CincD	QuinD	SIR + CincD	SIR + QuinD	HU
2	$\beta^0$39/ β^+IVSI-110	26.52	30.93	28.17	30.70	30.27	32.70	29.97
7	$\beta^0$39/ β^+IVSI-110	19.27	26.03	22.32	23.34	23.56	23.72	25.87
8	β^+IVSI-110/ β^+IVSI-110	8.32	13.4	12.2	15.06	18.14	17.62	12.61
9	$\beta^0$39/ $\beta^0$39	30.73	34.54	36.81	42.47	43.74	42.30	31.29
10	$\beta^0$39/ β^+IVSI-110	12.75	9.83	21.62	27.80	29.93	40.25	11.65

NT = untreated ErPCs; SIR, sirolimus; HU, hydroxyurea. Experimental conditions are reported in Figure 7.

Supplementary Table S3. Effects of cinchonidine (CincD) and quinidine (QuinD) on accumulation of free α-globin chains by ErPCs from β-thalassemia patients

patients	genotype	α-globin peak (% of total hemoglobins produced)				
		-	SIR	CincD	QuinD	HU
1	$\beta^0$39/ β^+IVSI-110	4.35	1.13	2.45	2.24	3.40
2	$\beta^0$39/ β^+IVSI-110	3.36	3.15	2.79	2.60	3.08
3	β^+IVSI-110/ β^+IVSI-110	3.14	1.70	1.86	1.66	4.24
4	$\beta^0$39/ β^+IVSI-110	8.23	6.97	2.56	2.97	8.38
5	$\beta^0$39/ $\beta^0$39	0.77	2.55	2.02	1.24	4.79
6	$\beta^0$39/ $\beta^0$39	6.18	3.33	1.34	1.56	3.80
7	$\beta^0$39/ β^+IVSI-110	3.34	2.78	2.65	2.76	2.83
8	β^+IVSI-110/ β^+IVSI-110	3.51	3.45	2.53	1.52	4.55
9	$\beta^0$39/ $\beta^0$39	10.23	7.99	5.34	2.12	7.49
10	$\beta^0$39/ β^+IVSI-110	1.20	0.79	0.49	0.45	0.39

NT = untreated ErPCs; SIR, sirolimus; HU, hydroxyurea. Experimental conditions are reported in Figure 8.
